# Intrapulmonary schwannoma presenting as an asymptomatic lung mass: a case report

**DOI:** 10.1186/s13256-023-04234-z

**Published:** 2023-12-11

**Authors:** Manzar Abbas, Asad Saulat Fatimi, Syed Imad Hassan, Faiqa Binte Aamir, Sidra Arshad, Saulat Hasnain Fatimi

**Affiliations:** 1 Medical College, Aga Khan University, Karachi, Pakistan; 2 Section of Histopathology, Department of Pathology and Laboratory Medicine, Aga Khan University, Karachi, Pakistan; 3 Section of Cardiothoracic Surgery, Department of Surgery, Aga Khan University, Karachi, Pakistan

**Keywords:** Neoplasm, Atypical schwannoma, Lobectomy

## Abstract

**Background:**

Schwannomas are solitary well-circumscribed encapsulated benign tumors that exhibit Schwann cell differentiation, and arise directly from myelinated peripheral or central nerves. Although they are usually asymptomatic and found incidentally, schwannomas can cause symptoms due to compression of nearby structures which, depending on the location, can make clinical presentations widely variable. Despite their rarity, schwannomas have been documented in a number of locations including the limbs, cerebellopontine angle, posterior mediastinum, and, far more infrequently, the lungs.

**Case presentation:**

In this article, we report an incidental finding of an intrapulmonary schwannoma in a 59-year-old Pakistani woman who was grossly asymptomatic upon presentation to the cardiothoracic surgery clinic. An [^18^F]fluorodeoxyglucose positron emission tomography/computed tomography scan revealed a lobulated soft-tissue lesion measuring 23 mm × 23 mm in the lower lobe of the right lung. A computed tomography-guided core biopsy of the mass was performed, which revealed a benign spindle cell lesion based on histopathological examination and immunohistochemical staining. The mass was surgically resected via a right lower lobectomy, and subsequently confirmed to be an encapsulated neoplastic lesion composed of well-differentiated Schwann cells. There were no short- or long-term complications, morbidities, or recurrences based on 1-year follow-up.

**Conclusion:**

This report underscores the predominantly asymptomatic nature of schwannomas and reemphasizes the efficacy of surgical resection as a safe and curative procedure for a tumor of this nature. Albeit very rare, intrapulmonary schwannomas can be considered a differential diagnosis when encountering solitary asymptomatic pulmonary nodules or masses.

**Supplementary Information:**

The online version contains supplementary material available at 10.1186/s13256-023-04234-z.

## Background

Schwannomas, also known as neurilemmomas or neurinomas of Verocay, frequently appear as solitary well-circumscribed encapsulated benign tumors that exhibit Schwann cell differentiation and arise directly from myelinated peripheral or central nerves [1]. The Schwann cell origin of these tumors is highlighted by the fact that they are immunoreactive to the peripheral nerve sheath tumor marker S-100 [1]. Grossly, these tumors appear as firm masses that may be round, ovoid, or lobulated with a soft-tissue density on a computed tomography (CT) scan [1]. Microscopically, they consist of a mixture of “dense” Antoni A areas containing spindle cells and palisading nuclei, and “loose” relatively hypocellular Antoni B areas that contain a prominent myxoid extracellular matrix [1].

Primary lung nerve cell tumors are incredibly uncommon, making up about 0.2% of all pulmonary neoplasms [2]. While approximately 90% of schwannomas are sporadic, some presentations may have syndromic associations, such as neurofibromatosis type 2 (NF2) and schwannomatosis [1, 3]. All schwannomas show diminished levels of the gene product of the *NF2* gene on chromosome 22, merlin, which is responsible for the regulation of several cell-signalling pathways by interacting with the actin cytoskeleton. As such, loss of merlin results in dysregulation of cell shape, cell growth, and cell-to-cell adhesion thereby leading to tumor formation [3].

Schwannomas have been described in a variety of locations, most commonly in the upper limbs and intracranially at the cerebellopontine angle [3]. They have also been described in intrathoracic locations including the posterior mediastinum, more specifically the costovertebral angle [2]. However, primary intrapulmonary tumors of this variety are extremely rare, as highlighted by the paucity of available literature.

In this article, we report the case of a 59-year-old woman who was incidentally diagnosed with an intrapulmonary schwannoma in the right lower zone of the right lung. This work is compliant with the 2020 Surgical CAse REport (SCARE) guidelines (Additional File 1) [4].

## Case presentation

A 59-year-old Pakistani woman with a past medical history of hypertension and a significant family history of cancer in first-degree relatives (breast, ovarian, and pancreatic cancer) was referred to the cardiothoracic surgery clinic. This was due to an incidental finding of a nodular density on her chest X-ray as part of a preoperative workup for a total left knee replacement due to osteoarthritis. However, there were no previous chest images for comparison. Aside from a minor complaint of blood-tinged sputum in the morning, there were no complaints or symptoms. She was vitally stable and saturating well on room air. Her general physical, cardiovascular, respiratory, abdominal, and central nervous system examinations were unremarkable.

**Fig. 1:**
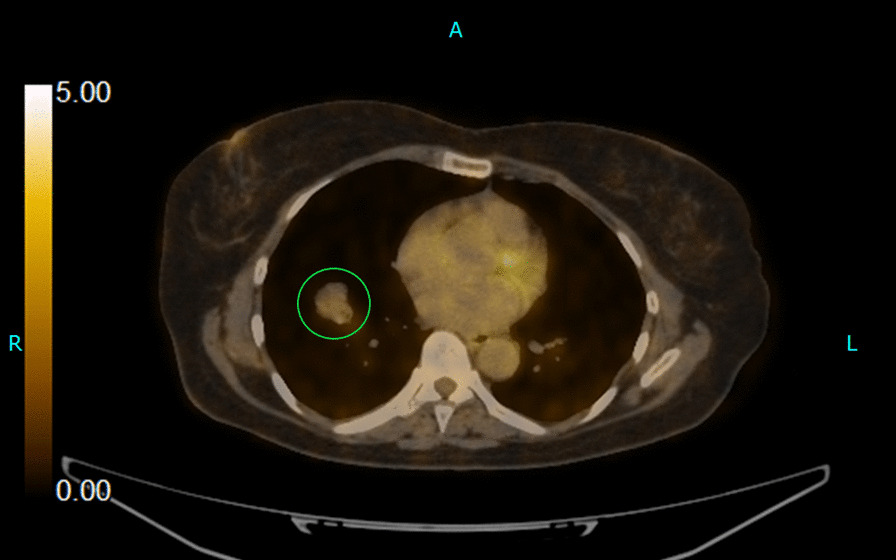
FDG PET/CT image; Lobulated soft tissue lesion in the right lung encircled in green

Radiological imaging (CXR) showed a nodular density in the right lower zone of the right lung measuring approximately 2.5 cm × 2 cm. A subsequently conducted high definition [^18^F]fluorodeoxyglucose (FDG) positron emission tomography/computed tomography (PET/CT) scan revealed a lobulated soft-tissue lesion measuring 23 mm × 23 mm (with internal cavitation) along the medial aspect of the anterior segment of the lower lobe of the right lung (Fig. 1). The lesion had a maximum standardized uptake value (SUV_max_) of 2.7. No evidence of hypermetabolic nodal, pulmonary, hepatic, splenic, adrenal, or bony metastasis was observed.

A CT-guided core biopsy of the lesion was performed. Histopathological evaluation revealed a core of a spindle cell lesion with smaller cores of native unremarkable lung parenchyma. Significant cytological atypia or increased mitotic activity was not seen. Immunohistochemical staining revealed the cells to be S100 focal positive, SOX-10 negative, ASMA negative, Melan A negative, HMB-45 negative, STAT6 negative, and CD34 patchy positive. A diagnosis of a benign spindle cell lesion, likely of neural origin, was made. An elective surgical resection was planned for a month later.

After standard preoperative optimization and preparation, a posterolateral thoracotomy with right lower lobectomy was performed under general anesthesia. The pleural cavity was entered through the fifth intercostal space. Enlarged station 9R and 10R lymph nodes and a mass in the lower lobe of the right lung were visualized, while no masses were observed in the middle or upper lobes. There were no pleural nodules or pleural effusion. The inferior pulmonary ligament was divided, and the inferior pulmonary vein was identified and divided between ties. The oblique fissure was incomplete, and was used to separate the upper, middle, and lower lobes of the lung through cautery. The pulmonary artery was identified at the confluence of the oblique and horizontal fissures, and the superior segmental and basilar branches were visualized. The superior segmental branch and the pulmonary artery distal to the take-off of the middle lobe branch were both divided between ties. The bronchus intermedius was dissected and the middle lobe bronchus was identified. The bronchus distal to the middle lobe take-off was dissected, and a TA-30 stapler was used to divide the superior segmental bronchus and the bronchus distal to the middle lobe take-off. The mass was then resected, and hemostasis was secured. The surgical wound was closed in layers after the placement of an extrapleural catheter and chest tubes, and the patient was shifted to the in-patient facility. The patient remained vitally and hemodynamically stable and was discharged 3 days after the operation. The patient was confirmed to be in her usual state of health upon 6-month and 1-year follow-up, with no late complications or recurrences.

**Fig. 2:**
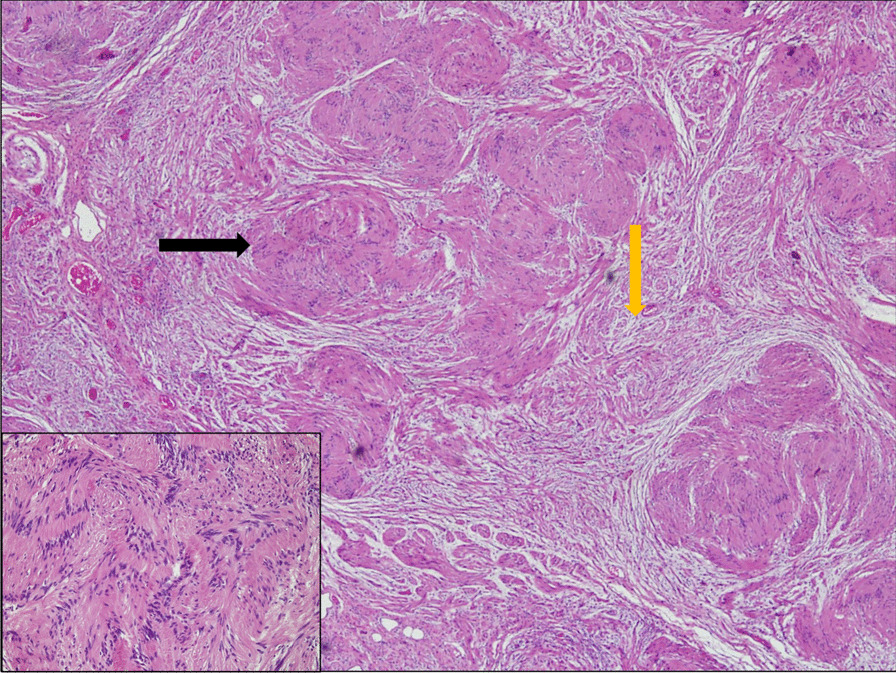
Histology of resected mass showing a neoplastic lesion composed of well-differentiated Schwann cells. Spindle-shaped cells ar shown at 20× magnification (bottom left), arranged in alternating hypercellular (Antoni A) and hypocellular (Antoni B) areas (yellow arrow). There is characteristic nuclear palisading around fibrillary process forming Verocay bodies (black arrow)

Histopathological examination of the resected lobe showed lung parenchyma infiltrated by an encapsulated neoplastic lesion composed of well-differentiated Schwann cells. The neoplastic cells showed a biphasic pattern with compact areas of spindle cells having moderate eosinophilic cytoplasm and normal chromatic elongated nuclei. Areas of occasional palisading (Verocay bodies) were also seen (Fig. 2). These morphological findings, combined with immunohistochemical staining results concurrent with those mentioned earlier, were consistent with the diagnosis of an intrapulmonary schwannoma.

## Discussion

Schwannomas present as well-circumscribed encapsulated masses and are solitary in up to 90% of cases [3]. Although they are usually asymptomatic, found incidentally (as was the case with our patient), and may exist for years without any clinical manifestations [3], schwannomas can cause symptoms due to compression of nearby structures. Moreover, due to the plethora of locations where these tumors may occur, clinical presentations are widely variable.

While the most common locations for a schwannoma are in the upper limb and the cranial vault (at the cerebellopontine angle, wherein the schwannoma is referred to as an “acoustic neuroma”) [3], the incidence of intrapulmonary schwannomas is extremely rare.

Radiological examinations usually reveal a round, ovoid, or lobulated mass with soft-tissue density and well-demarcated borders [5], as was seen in our patient who had a solitary nodular density on CXR. Moreover, our patient had an SUV_max_ of 2.7, which is concurrent with existing literature that suggests a value between 1.3 and 6 for benign neurogenic tumors [6]. However, while FDG PET/CT has shown potential in differentiating between malignant peripheral nerve sheath tumors (MPNST) and neurofibromas [7], the utility is limited for schwannomas given the great degree of variability in the SUV_max_ value due to microvascular and cellular density, and a significant proportion of schwannomas being highly metabolic variants with an SUV_max_ of > 6 [6, 7]. As such, a biopsy must be done for confirmatory diagnosis.

Histopathological examination of the biopsied schwannoma will reveal compact eosinophilic spindle cell areas (Antoni A areas) and hypocellular areas with an abundant extracellular matrix (Antoni B areas) [1], as in our case. However, given that spindle cells are present in a variety of neurogenic tumors, immunohistochemical staining with markers including S-100, SMA, CD34, and CD31 is necessary to conclusively diagnose a schwannoma.

Given these tumors are loosely attached to their associated peripheral or central nerves [1], they can be surgically resected without sacrificing the nerve. The location of the tumor in the lung parenchyma in our case necessitated a lower lobe lobectomy of the right lung. However, incomplete surgical resection may lead to a recurrence in up to 5% of cases [1], although there is little risk of malignancy, unlike plexiform neurofibromas, which are associated with neurofibromatosis type-1 (NF1) [1]. Treatment techniques for intrapulmonary schwannomas have also included intrabronchial resection with endoscopy and yttrium aluminium garnet (YAG) laser resection [5]. Documented cases of intrapulmonary schwannoma show very favorable prognoses, as observed in our patient who developed no short-term or late-term complications or morbidities.

## Conclusion

In conclusion, this report highlights the rare case of an intrapulmonary schwannoma and underscores the predominantly asymptomatic nature of such tumors, which are often discovered incidentally during radiographic examinations performed for unrelated reasons. The successful surgical excision performed in this case demonstrated its efficacy as a safe and curative procedure, with no observed short-term or late-term complications or morbidities. These findings align with existing literature and emphasize that, albeit very rare, intrapulmonary schwannomas can be considered a differential diagnosis when encountering solitary asymptomatic pulmonary nodules or masses.

### Supplementary Information

**Additional file 1.** Completed SCARE Checklist for the Present Article.

## Data Availability

Not applicable.
